# The Papez Circuit in First-Episode, Treatment-Naive Adults with Major Depressive Disorder: Combined Atlas-Based Tract-Specific Quantification Analysis and Voxel-Based Analysis

**DOI:** 10.1371/journal.pone.0126673

**Published:** 2015-05-21

**Authors:** Wenyan Jiang, Gaolang Gong, Feng Wu, Lingtao Kong, Kaiyuan Chen, Wenhui Cui, Ling Ren, Guoguang Fan, Wenge Sun, Huan Ma, Ke Xu, Yanqing Tang, Fei Wang

**Affiliations:** 1 Department of Psychiatry, The First Affiliated Hospital, China Medical University, Shenyang 110001, Liaoning, PR China; 2 Department of Radiology, The First Affiliated Hospital, China Medical University, Shenyang 110001, Liaoning, PR China; 3 Department of Psychiatry, Yale University School of Medicine, New Haven, Connecticut, United States of America; 4 Department of Radiology, The Liaoning Cancer Hospital & Institute, Shenyang 110042, Liaoning, PR China; 5 State Key Laboratory of Cognitive Neuroscience and Learning, Beijing Normal University, Beijing, 100875, PR China; Hangzhou Normal University, CHINA

## Abstract

Previous findings suggest that the Papez Circuit may have a role in major depressive disorders. We used atlas-based tract-specific quantification analysis and voxel-based analysis to examine the integrity of white matter tracts involved in mood regulation (including tracts in the Papez Circuit). Diffusion tensor imaging acquired from 35 first-episode, treatment-naive adults with major depressive disorders and 34 healthy adult controls were compared. Our statistical approach compared structural integrity of 11 major white matter tracts between the major depressive disorder and adult controls, as well as illness duration influence in patients. Fractional anisotropy was decreased in the hippocampal cingulum and in the anterior thalamic radiation according to both analytical approaches, all of which were important tracts included in the Papez Circuit. Our results support the role of the Papez Circuit in major depressive disorders with the minimal probability of false positive due to similar findings in both analyses that have complementary advantages. Dysfunction of the Papez Circuit may be a potential marker for studying the pathogenesis of major depressive disorders.

## Introduction

Major depressive disorder (MDD)—a common disorder with a chronic and recurring pattern and a lifetime prevalence of 16.2% [1] is a leading cause of disability worldwide [2]. Magnetic resonance imaging (MRI) provides a noninvasive means to measure structural differences among individuals and has significantly advanced our understanding of the neuropathophysiology of MDD. Initial studies have chiefly focused on the use of structural magnetic resonance imaging (sMRI), but with the discovery of pulsed sequence measurements, such as functional MRI (fMRI) and diffusion tensor imaging (DTI), not only has regional dysregulation been discovered but also the disruption of a neural functional network has been identified [3], [4], [5].

The Papez circuit was postulated by James Papez to be like many other areas of the limbic system involved in emotion. Papez originally speculated that this comprised the anatomical substrate of emotional experience. As one of two major pathways into and out of the hippocampus (the other being the entorhinal cortex, via the cingulate cortex), the fornix connects to the hippocampus and the mammillary bodies of the hypothalamus, and it enters the anterior thalamic nucleus via the mammillothalamic tract. The anterior thalamic nuclei, in turn, connect to the cingulate cortex, which projects back to the entorhinal cortex of the parahippocampal gyrus, completing the Papez circuit [6], [7]. We found that the limbic system (Papez circuit) is activated when emotion is evoked. Emotions are mostly mediated through the Papez circuit of the limbic system to determine the final expression of emotions [8]. Papez circuit disruption is associated with affective processing and cognitive functioning disturbances [9], [10]. It has also been proposed that the Papez circuit is related to depression symptoms [11]. Additional evidence from MRI studies further supports the involvement of the Papez circuit in the pathophysiology of MDD. For example, altered grey and white matter (WM) within those regions (such as the cingulate, hippocampus and parahippocampal gyrus) were observed in MDD subjects [12], [13], [14], in our study [15], [16], [17]. Taken together with the findings from fMRI [18], [19], abnormalities of WM tracts related to the dysfunction of the Papez circuit may be directly relevant to the pathophysiology of MDD.

DTI is an MRI technique that can provide information about structural integrity of WM tracts in vivo by measuring the magnitude and direction of water diffusion. Specifically, DTI has been used successfully to investigate WM abnormalities in MDD [20]. Two DTI processing methods, atlas-based tract-specific quantification analysis [21] and whole-brain voxel-based analysis (VBA) [22], have been used in recent studies, and have greatly enhanced the understanding of the structure-function relationship in the human brain. Although it is useful for us to identify changes in WM tracts, atlas-based tract-specific quantification analysis is limited in the selection and placement of region(s) of interest (ROI) [23]. VBA, a more global and automated exploratory strategy that can direct further detailed analyses by testing each voxel for statistical differences between population cohorts, also depends on the selection of different settings in VBA processing [24]. The combination of ROI and whole-brain analysis [23], [25] is effective for measuring the microstructural integrity of neuronal tracts, so this method can be used to explore the hypothesis that MDD is associated with a disruption of neural connectivity. For this purpose, atlas-based tract-specific quantification analysis and VBA were both employed to examine the integrity of WM tracts involved in mood regulation (including the tracts in the Papez Circuit) in our study. Our report is the first, to our knowledge, to use first-episode, treatment-naive adults with MDD. Analysis based on an anatomical atlas may be suitable for avoiding both intra- and inter-rater variability in manual delineation of ROI, as well as assuring anatomical location. Therefore, our primary objective was to define the role of dysfunction in specific networks of the Papez circuit in MDD, as measured by DTI. We hypothesize that damage of WM tracts involved in the Papez Circuit are associated with the pathogenesis of MDD.

## Materials and Methods

### Participants

We enrolled patients with MDD from the outpatient clinics at the Department of Psychiatry, at the First Affiliated Hospital of China Medical University. All patients were diagnosed individually by two trained psychiatrists using the Structured Clinical Interview for DSM-IV and fulfilling DSM-IV [26] criteria for major depressive disorder. Patients who met the following inclusion criteria were enrolled: having had a first depressive episode; aged 18 to 46 years; no comorbid DSM-IV Axis I or II diagnosis; having a score of at least 17 on the 17-item Hamilton Depression Rating Scale (HDRS-17) [27]; and no history of psychotropic medication use, electroconvulsive therapy or psychotherapy prior to MRI scanning. Clinical assessment included the HDRS and the Hamilton Anxiety Rating Scale (HARS) [28].

We also recruited healthy control subjects from the community. Individuals with an absence of DSM-IV Axis I disorders in their personal history or their first-degree family members were included. Control participants were confirmed for enrollment using the Structured Clinical Interview for DSM-IV Disorders.

Exclusion criteria for all participants included the following: 1) any MRI contraindications; 2) history of head trauma with loss of consciousness greater than 5 min or any neurological disorder; 3) any concomitant major medical disorder; 4) substance abuse or dependence within the last 6 months. All participants were right-handed and scanned within 48 h of initial contact. Written informed consent was obtained from all participants after they have been offered a detailed description of the study. The study was approved by the Institutional Review Board of the China Medical University.

### Ethics statement

The study was approved by the Institutional Review Board of the China Medical University. All participants provided their written informed consent after detailed description of the study.

### MRI Acquisition

Diffusion-weighted images were acquired on a 3T MR scanner (General Electric, Milwaukee, WI) at the First Affiliated Hospital of China Medical University, Shenyang, China. Head motion was minimized with restraining foam pads. A standard head coil was used for radiofrequency transmission and reception of the nuclear magnetic resonance signal. Diffusion tensor images were acquired using a spin-echo planar imaging sequence, parallel to the anterior-posterior (AC-PC) plane. The diffusion sensitizing gradients were applied along 25 non-collinear directions (b = 1,000 s/mm^2^), together with an axial acquisition without diffusion weighting (b = 0). These were the scanning parameters: repetition time (TR) = 17,000 ms; echo time (TE) = 85.4 ms; field of view (FOV) = 240×240 mm^2^; image matrix = 120×120; 65 contiguous slices of 2 mm without gaps.

### DTI Processing

**Fig 1:**
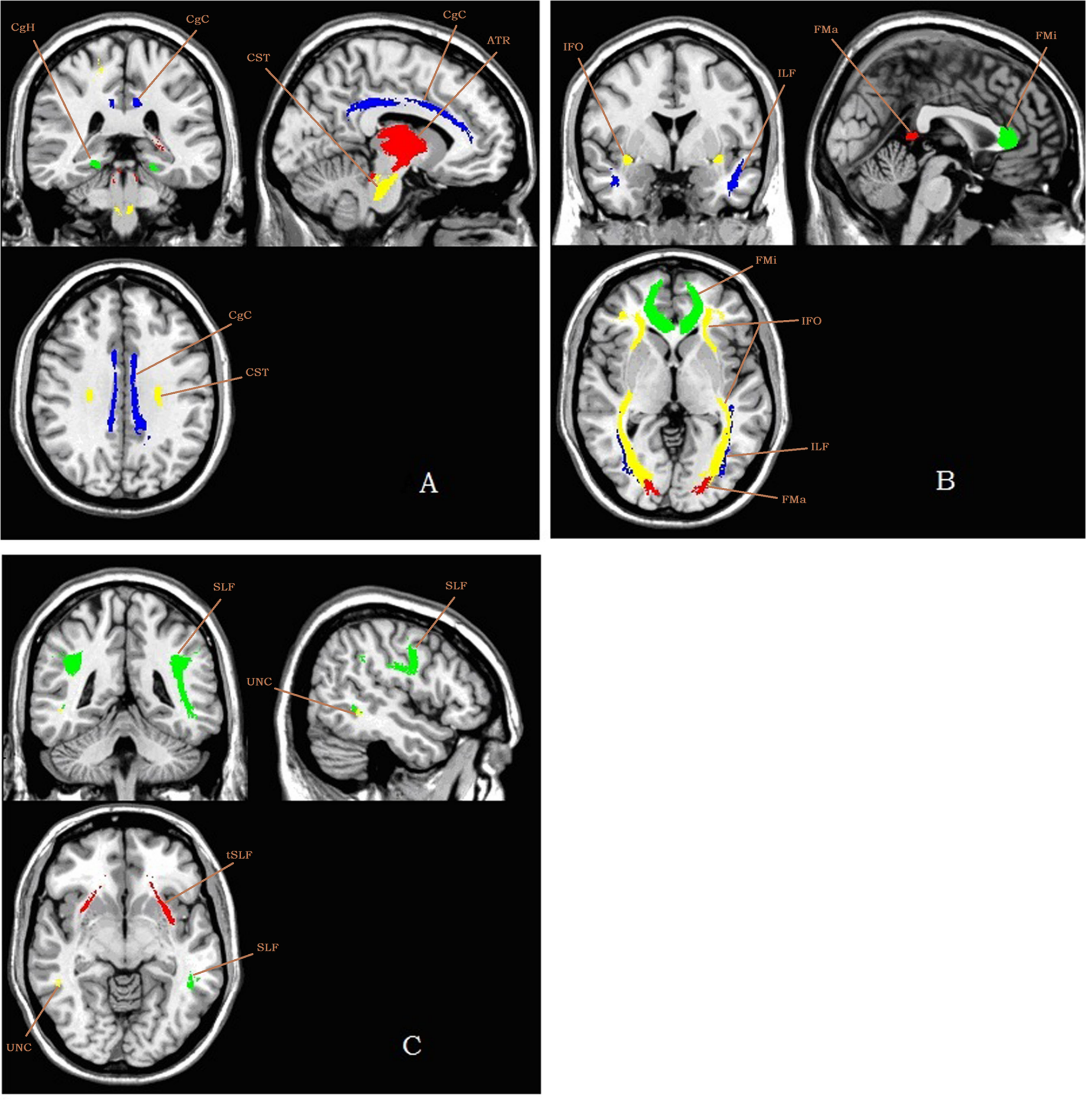
11 fiber tracts in atlas-based tract-specific quantification analysis superimposed on a ch2 template. Abbreviations: A: ATR, anterior thalamic radiation (red); CST, corticospinal tract (yellow); CgC, cingulum in the cingulate cortex area (blue); CgH, cingulum in the hippocampal area (green); B: FMa, forceps major (red); FMi, forceps minor (green); IFO, inferior fronto-occipital fasciculus (yellow); ILF, inferior longitudinal fasciculus (blue); C: SLF, superior longitudinal fasciculus (green); tSLF, the temporal projection of the SLF (red); UNC, uncinate fasciculus (yellow).

First, diffusion-weighted images were analyzed using FSL (FMRIB Software Library, http://www.fmrib.ox.ac.uk/fsl/). Several FSL tool packages were used for data processing. After motion and eddy current corrections, fractional anisotropy (FA) images were created by fitting a tensor model to the raw diffusion data using FDT (FMRIB's Diffusion Toolbox), and skull-stripping using BET (Brain Extraction Tool). All subjects' FA data were then aligned into a 1×1×1 mm^3^ standard space image (MNI152 space) using the nonlinear registration tool FNIRT (FMRIB's Non-Linear Image Registration Tool), which uses a b-spline representation of the registration warp field, and a mean FA image was created from images of all subjects [29], [30]. A FA threshold of 0.2 was used in both VBM and atlas-based tract-specific quantification analyses to reduce the effect of other brain tissues. In the first approach, we identified the anatomical regions to measure using a probabilistic WM tract atlas including 11 fiber tracts (Fig 1; all are present bilaterally except for forceps major [FMa] and forceps minor [FMi]) developed from the manually traced maps of 27 neurologically normal subjects (atlas provided by S. Mori and the Johns Hopkins Medical Institute Laboratory of Brain Anatomical MRI) [31]. The White Matter Parcellation Map (WMPM) was consulted for these slices and served as a guide for shapes and sizes of ROIs. Then, the WMPM was automatically applied to the normalized images, and FA values of the WM fiber tracts were measured. An automated tract-specific quantification of FA approach was performed with MriStudio/RoiEditor (www.MriStudio.org or mri.kennedykrieger.org) [32]. In the second approach, voxel-based analysis of the diffusion tensor was performed with Statistical Parametric Mapping 5 (SPM5) (http://www.fil.ion.ucl.ac.uk/spm). The normalized images were resampled with a final voxel size of 2×2×2 mm^3^ and spatially smoothed with a 6-mm full width half maximum Gaussian kernel to reduce effects of misalignment caused by imperfect registration. Then, voxelwise t-statistic for between-group comparison was done and the results were overlapped on the MIN152 template and Hopkins probabilistic WM tract atlas [31] in MRIcro (http://www.mccauslandcenter.sc.edu/CRNL/tools).

### Statistical Analysis

Independent-sample t-tests and χ^2^ tests were used to compare demographic data and HDRS scores between the groups with SPSS for Windows software, version 18.0 (SPSS Inc., Chicago, IL, 2009). FA values of tract-specific quantification analyses were also conducted using SPSS18.0. Spearman correlation analyses were performed to test the relationships between FA values and illness duration within the MDD sample. Two-sample t tests were performed in a voxel-by-voxel manner with SPM5. To decrease the chance of false positive, results were considered statistically significant at the height threshold of P<0.001 (two-tailed, uncorrected) in VBM and P<0.01 (two-tailed, uncorrected) in atlas-based tract-specific quantification analysis, as well as a cluster-extent threshold of 10 voxels throughout.

## Results

### General Data Analysis

We enrolled 35 patients with MDD (mean 31.91 ± 8.80 years-of-age, 18 females) and 34 healthy control subjects (mean 29.54 ± 8.57 years-of-age, 17 female). There were no significant differences between the MDD and adult control (HC) subjects with regard to age, gender, and education (P>0.05). The MDD group had significantly higher HDRS and HARS scores than the HC group (P<0.001). In patients, the duration of illness had no relation with the mean HDRS score (P>0.05) and there were no differences in age, gender, and education (P>0.05). Detailed demographic and clinical data of participants appears in Table 1.

**Table 1:** Subject Demographic and Clinical Data.

	HC	MDD
**Number**	34	35
**Age (years, mean ± SD)**	31.91 ± 8.80	29.54 ± 8.57
**Sex (male:female)**	17:17	17:18
**Education (years, mean ± SD)**	14.57 ± 3.07	13.20 ± 2.78
**HDRS (mean ± SD)**	0.79 ± 1.21	27.70 ± 5.21
**HARS (mean ± SD)**	0.28 ± 0.70	20.50 ± 7.86
**Duration of illness (months, mean ± SD)**	N/A	12.83 ± 17.02

MDD, major depressive disorder; SD, standard deviation; HDRS, Hamilton Depression Rating Scale; HARS, Hamilton Anxiety Rating Scale.

### Atlas-based Tract-specific Quantification Analysis

The mean FA values in the WM tracts are shown in Table 2.

**Table 2:** Cohort Differences of Mean FA Values in the WM Tracts.

WM tracts	HC	MDD	t value	p value
**Anterior thalamic radiation L**	0.401 ± 0.014	0.395 ± 0.014	1.803	0.076
**Anterior thalamic radiation R**	0.375 ± 0.014	0.366 ± 0.014	2.785	0.007
**Corticospinal tract L**	0.542 ± 0.015	0.547 ± 0.019	−1.067	0.290
**Corticospinal tract R**	0.553 ± 0.017	0.556 ± 0.022	−0.762	0.449
**Cingulum (cingulate gyrus) L**	0.433 ± 0.022	0.431 ± 0.026	0.435	0.665
**Cingulum (cingulate gyrus) R**	0.401 ± 0.021	0.395 ± 0.027	1.026	0.309
**Cingulum (hippocampus) L**	0.336 ± 0.022	0.327 ± 0.028	1.535	0.129
**Cingulum (hippocampus) R**	0.371 ± 0.026	0.352 ± 0.026	3.016	0.004
**Forceps major**	0.529 ± 0.018	0.437 ± 0.015	1.015	0.314
**Forceps minor**	0.444 ± 0.016	0.437 ± 0.018	1.127	0.264
**Inferior fronto-occipital fasciculus L**	0.436 ± 0.013	0.430 ± 0.019	1.722	0.090
**Inferior fronto-occipital fasciculus R**	0.383 ± 0.014	0.381 ± 0.017	1.406	0.165
**Inferior longitudinal fasciculus L**	0.047 ± 0.015	0.404 ± 0.021	0.695	0.489
**Inferior longitudinal fasciculus R**	0.378 ± 0.013	0.378 ± 0.014	0.781	0.438
**Superior longitudinal fasciculus L**	0.390 ± 0.015	0.391 ± 0.014	0.061	0.951
**Superior longitudinal fasciculus R**	0.375 ± 0.014	0.375 ± 0.018	−0.323	0.748
**Uncinate fasciculus L**	0.410 ± 0.022	0.402 ± 0.024	1.440	0.155
**Uncinate fasciculus R**	0.414 ± 0.027	0.406 ± 0.024	1.172	0.245
**Superior longitudinal fasciculus (temporal part) L**	0.467 ± 0.017	0.468 ± 0.018	−0.164	0.870
**Superior longitudinal fasciculus (temporal part) R**	0.468 ± 0.023	0.473 ± 0.026	−0.835	0.407

WM, white matter; HC, adult control; MDD, major depressive disorder.

Compared with the HC group, the MDD group had lower FA values in the right anterior thalamic radiation (ATR) (t = 2.785, p<0.01) and the right cingulum in hippocampus (CgH) (t = 3.016, p<0.01). Among patients, none of FA values in WM tracts was significantly associated with duration of illness (p>0.05). HDRS and HARS scores and age and gender were not related to FA values either (p>0.05).

### VBA

**Fig 2:**
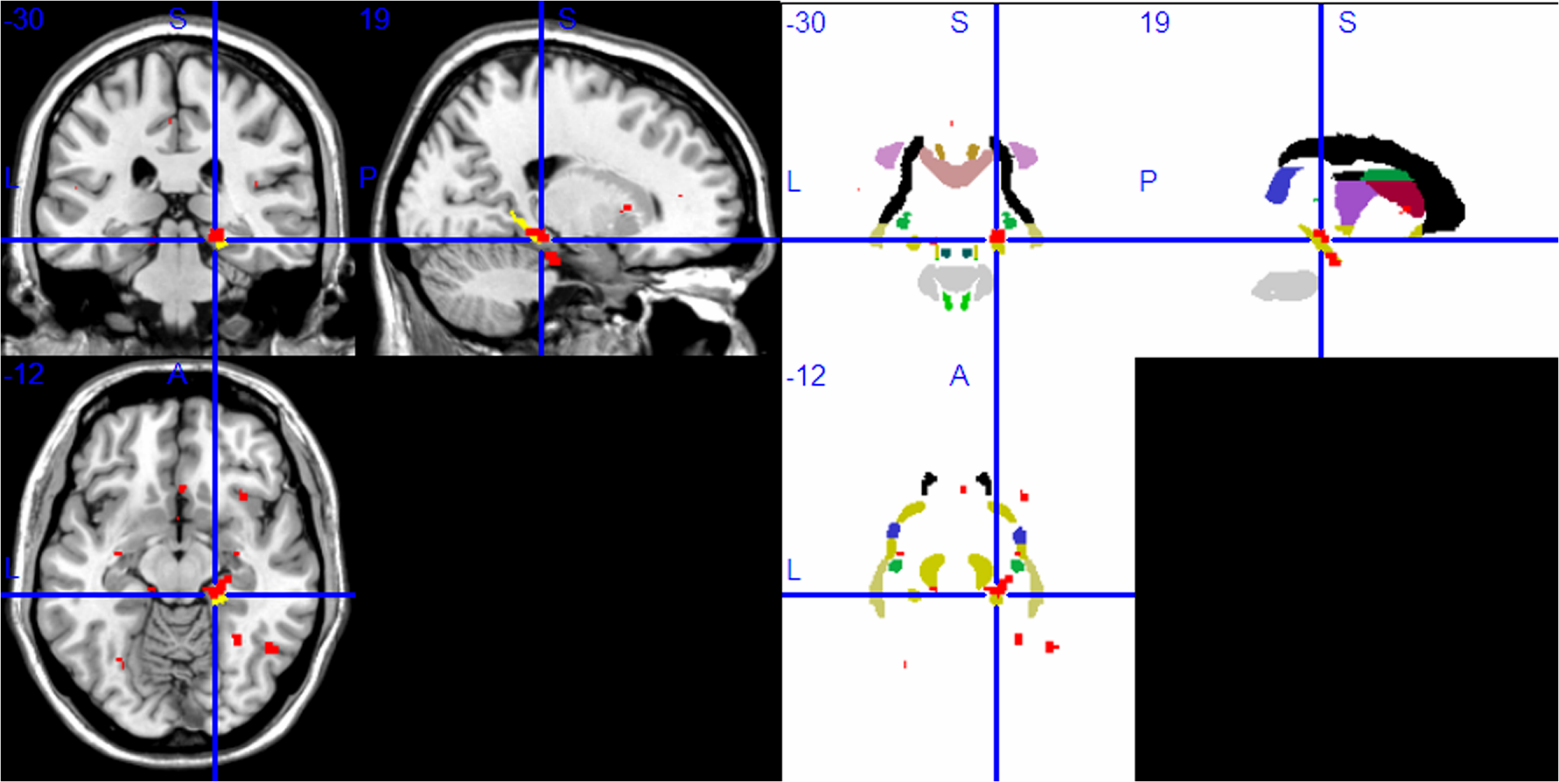
Compared with the HC group, the MDD group had lower FA values in the right Parahippocampal gyrus in voxel-based diffusion tensor analysis (A:red; B: red in superimposed on WM tracts template). Abbreviation: HC, healthy control; MDD, major depressive disorder; FA, fractional anisotropy; WM, white matter.

**Fig 3:**
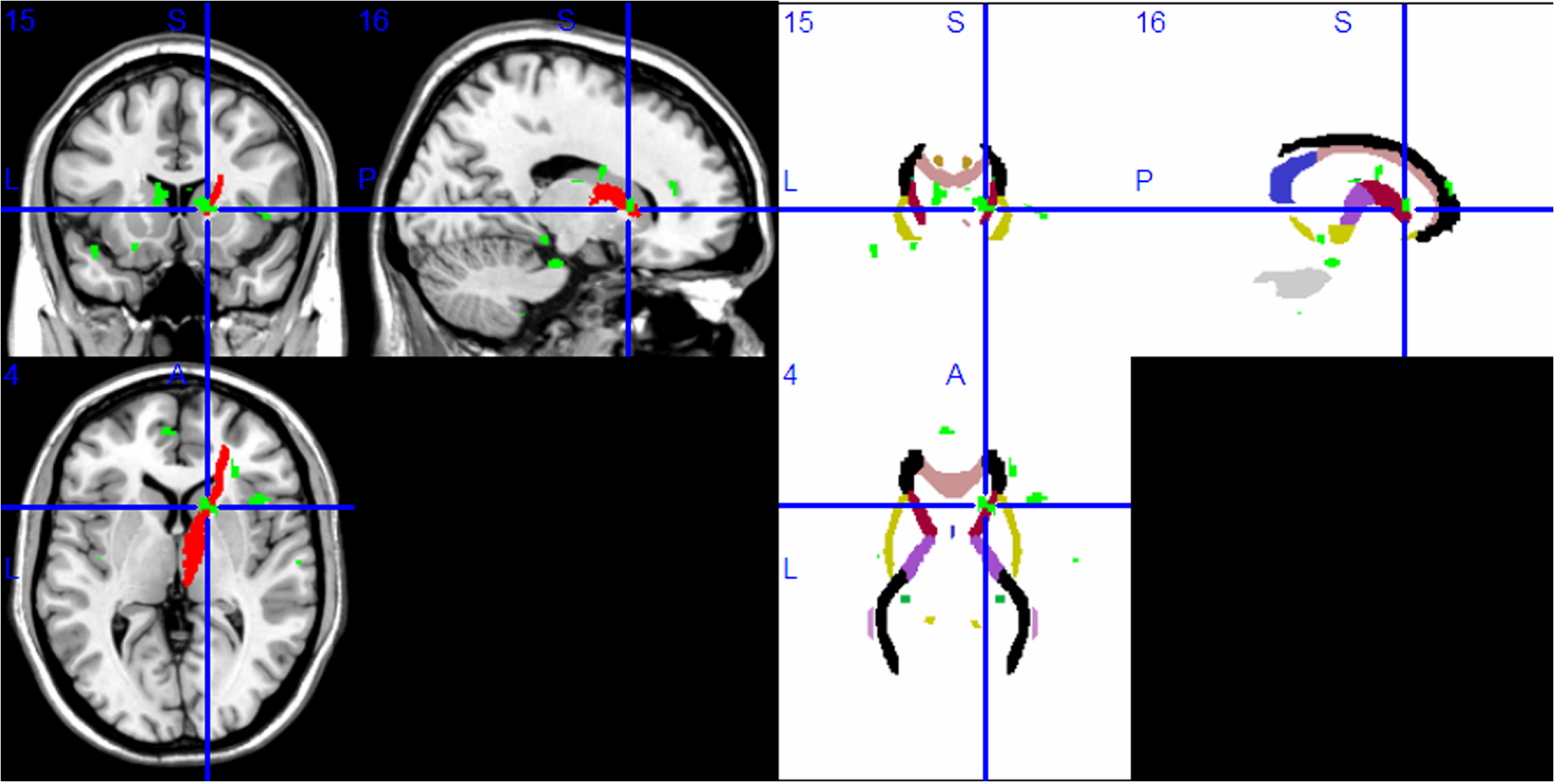
Compared with the HC group, the MDD group had lower FA values in the right anterior thalamic radiation in voxel-based diffusion tensor analysis (A: green; B: green in superimposed on WM tracts template). Abbreviation: HC, healthy control; MDD, major depressive disorder; FA, fractional anisotropy; WM, white matter.

**Fig 4:**
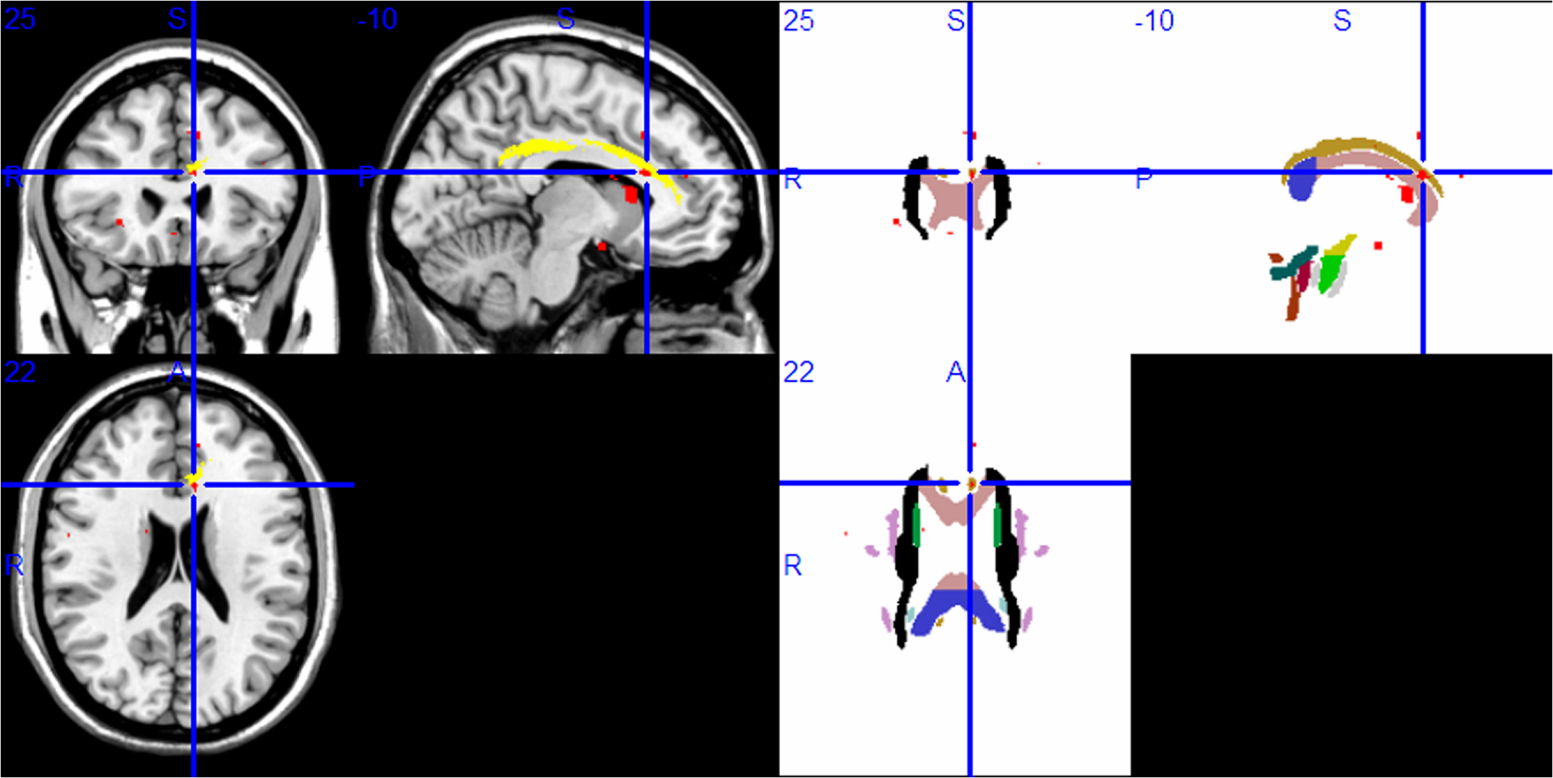
Compared with the HC group, the MDD group had lower FA values in the left anterior cingulate (A: red; B: red in superimposed on WM tracts template). Abbreviation: HC, healthy control; MDD, major depressive disorder; FA, fractional anisotropy; WM, white matter.

WM areas of the Papez circuit had lower FA values in the right parahippocampal gyrus (Fig 2), the right ATR (Fig 3), and the left anterior cingulate (Fig 4) (P<0.001, uncorrected). Lower FA values were also found in other brain regions (P<0.001, uncorrected): bilateral inferior frontal gyrus, right middle frontal gyrus, right middle occipital gyrus, right insula, and left caudate body. Table 3 lists all of the brain regions with statistically significantly reduced FA values for the MDD sample in contrast to the HC sample.

**Table 3:** Comparison of FA Values in MDD and HC (Compared with MDD, Uncorrected).

Brain regions with decreased FA values	voxel	MNI co-ordinate(x, y, z)	t value
**Left inferior frontal gyrus**	14	−36, 20, −8	4.10***
**Right inferior frontal gyrus**	24	52, 10, 16	4.68***
**Right middle frontal gyrus**	22	26, 52, 12	4.36***
**Right middle occipital gyrus**	18	48, −60, −10	4.68***
**Left anterior cingulate**	24	−2, 28, 19	4.11***
**Right parahippocampal gyrus**	56	20, −30, −10	3.99***
**Right anterior thalamic radiation**	49	12, 12, 6	4.69***
**Right insula**	64	34, 20, 10	4.94***
	17	36, −12, 10	4.28***
**Left caudate body**	91	−8, 18, 10	4.23***

FA, fractional anisotropy; MDD, major depressive disorder; HC, healthy control.

***P < 0.001.

## Discussion

Using complementary atlas-based tract-specific quantification analysis and VBA, the integrity of WM tracts involved in mood regulation (including tracts in the Papez Circuit) were investigated. Lower FA values were found in the ATR, the CgH (WM in the parahippocampal gyrus), and the cingulum of the cingulate cortex area (WM in the cingulate cortex area) in participants with MDD; these tracts are included in the Papez Circuit. In addition, decreased FA values in WM tracts were not correlated with patient demographics and/or severity of illness. Complementary ROI- and VBA DTI methods yielded consistent results [33]. Since both VBA and atlas-based tract-specific quantification statistical analyses were applied to the same cohort, they should yield similar results. Inconsistent results could indicate a false positive, caused by limitations of the analysis methods. ROI-based DTI permitted testing in specific WM tracts and minimized the risk of a type I error, whereas VBA DTI permitted further localization within homologous regions. Taken together, these findings suggest WM abnormalities in the Papez Circuit identified by DTI may play an important role in the pathogenesis of MDD, and that this difference may not be due to illness progression or severity.

To our knowledge, this study provides the first evidence of WM tract abnormalities in the Papez Circuit in a sample of medication-naive adults with MDD according to atlas-based tract-specific quantification analysis and VBA. Additionally, most participants had relatively short illness durations (12.83 ± 17.02 months). Therefore, our findings were not influenced by treatment, and chronicity-related confounds were minimized.

A major component of the Papez circuit, the cingulum, has long fibers providing frontotemporal connections and shorter fibers connecting adjacent portions of the cingulate cortex [34]. Via the CgH (WM in the parahippocampal gyrus) and the precommissural branch of the fornix, the cingulum (anterior cingulum especially, as it provides substantial connections from the anterior cingulate cortex to the orbitofrontal cortex, and to mesial temporal and striatal structures [34]) is connected with the hippocampus, another component of the Papez circuit. It is established that hippocampal function is important for the pathogenesis of MDD [3, 35]. On the other hand, the cingulate cortex, which also shares extensive connections with the amygdale [36] and regulates its response (essential to emotional processing) [37], can be regulated by the anterior thalamic nuclei via the anterior thalamic radiation. Destruction of these three major WM tracts of the Papez circuit were observed in this work, and these data confirm those of Zhu’s group who reported decreased FA values in three WM tracts: the anterior limb of the internal capsule (composed of the frontopontine tract and anterior thalamic radiations), the parahippocampal gyrus, and the posterior cingulate cortex [38]. These results were consistent with findings from a recently published study about regional dysfunction of the Papez circuit, such as the cingulate cortex [39] and hippocampus [3], [35], [40]; inconsistent findings in other studies [40], [41] may be explained by disruptions of a neural functional network, not only regional dysregulation.

With previous neuroimaging studies, decreased gray matter in the anterior cingulate and hippocampus, as well as the parahippocampal gyrus was observed in MDD [14], [15]. Compared with data obtained with other methods, DTI studies of MDD confirm WM abnormalities. Pilot studies of DTI in MDD mainly suggest frontal gyrus (superior, middle, inferior), parahippocampal gyrus, anterior cingulate cortex, hippocampus, corpus callosum and insula [42], [43],[44] had regional WM abnormalities. Our findings extend the current body of evidence to suggest that WM abnormalities exist in these regions of the Papez circuit in patients with MDD and that these are accompanied by substantial abnormalities in the major WM tracts of the Papez circuit which connects them. Moreover, given the role of the Papez circuit in subserving emotional regulation, our findings may illuminate mechanisms underlying circuitry dysfunction in the Papez circuit that contribute to emotional dysregulation. Not only useful for MDD patients, such data may be used to improve the quality of life in other patients. The latest research indicates that neuropsychiatric symptoms caused by WM damage occur after chemotherapy in brains of tumor patients [45], and WM FA can be used to help rehabilitate function after therapy [46]. Thus retention of the integrity of most major WM tracts of the Papez circuit may help patients avoid depression and other psychiatric symptoms arising from illness or therapy and improve their quality of life.

Lower FA values were found in other brain regions: bilateral inferior frontal gyrus, right middle frontal gyrus, right middle occipital gyrus, right insula, and the left caudate body, and these are consistent with previous studies [42], [43], [44] and our work [16]. However, some data differ such as findings within the superior frontal gyrus [47]. In addition, other tracts implicated in MDD, such as the superior longitudinal fasciculus and the uncinate fasciculus which were significantly positively associated with depression severity [48], [49] were not confirmed in our study. Also, the cingulum in the cingulate cortex is an area that provides connectivity between the anterior cingulate and insula [50], did not appear to have lower FA in our study. We speculate that perhaps dysfunction in the Papez Circuit is essential for MDD rather than merely a component.

Our study was limited with respect to some methodological aspects. First, as members of WM tracts in the Papez circuit, the fornix and mammillothalamic tract were not investigated because the atlas used for defining the ROI was limited in atlas-based tract-specific quantification analysis. Next, our results were mainly detected in young adults with MDD, no restriction on duration of illness in our current study. So, pathophysiology varies according to age and duration of illness might exist in MDD patients. But, patients that we enrolled having a score of at least 17 on the HDRS-17, and the score was (27.70 ± 5.21) finally. The result that the role of the Papez Circuit in major depressive disorders come from a group of MDD patients seriously and typically. The changes can be revised by treatment [51]. Therefore, we think the Papez circuit may still be a potential maker of the disease in spite of no association between the circuit and disease symptoms/severity in our study. We hypothesize that Papez circuit dysfunction may be not a “state” marker but a “trait” marker.

Further work is necessary to differentiate alterations in WM integrity related to MDD from those resulting from a more comprehensive atlas. Also, a larger sample size and a stricter significance threshold, as well as a sample that allows for comparisons across duration of illness may offer more convincing findings.

We observed abnormalities in major WM tracts of the Papez circuit in medication-naive MDD patients and these data support a role for the Papez Circuit in MDD. When combined with data from previous studies, our work suggests that dysfunction of the Papez Circuit may be important to the pathophysiology of MDD. Future studies are warranted to elucidate a neurodevelopmental mechanism contributing to the disorder.
